# Supplementary material for: The School Attachment Monitor—A novel computational tool for assessment of attachment in middle childhood
Source: PLoS One. 2021 Jul 22;16(7):e0240277. doi: 10.1371/journal.pone.0240277 (PMC8297900; doi:10.1371/journal.pone.0240277)
Supplement: S1 Fig — Parental consent was obtained for the use of the image. (DOCX) [file pone.0240277.s001.docx]

**S1 Fig – Computerised MCAST**

*Minnis et. al. International Journal of Methods in Psychiatric Research. 2010 2010;19(4):233-41.*

Parental consent was obtained for the use of the image
