# Supplementary material for: The School Attachment Monitor—A novel computational tool for assessment of attachment in middle childhood
Source: PLoS One. 2021 Jul 22;16(7):e0240277. doi: 10.1371/journal.pone.0240277 (PMC8297900; doi:10.1371/journal.pone.0240277)
Supplement: S1 Appendix — (DOCX) [file pone.0240277.s002.docx]

**S1 Appendix**

The automatic approach used in the experiments is fully described in Roffo et al. (2019) [[34](#_ENREF_34)]. The first step of the approach is to convert video recordings of children undergoing the MCAST into sequences $X=(\bar{x}_{1},\bar{x}_{2},\ldots,\bar{x}_{T})$ where every element is a vector that corresponds to a frame ($T$ is the total number of frames). Such a representation is suitable for computer processing and, in particular, for the application of Long Short Term memory networks [[40](#_ENREF_40)]. Every vector $\bar{x}_{k}$accounts for the pose that a child being recorded shows in frame $k$. The pose is extracted automatically with OpenPose, a publicly available software package that provides the position of the joints of every person depicted in an image [[35](#_ENREF_35)]. The components of the vectors $\bar{x}_{k}$, referred to as features hereafter, are 11 and account for the following properties of the pose (they can be obtained by simply processing the positions of the joints):

- Hand positions (4 features);
- Distance between hands (1 feature);
- Hands speed (2 features);
- Hands acceleration (2 features);
- Hands $1D$ trajectories (2 features);

The $1D$ trajectories were obtained by calculating the number $t=x+wy$, where $w$ is the width of the frames. All features where normalized through a $z$-transform, meaning that they were reduced by their average (so that the mean of the transformed features is $0$) and divided by their standard deviation (so that the standard deviation of the transformed features is $1$).

The vector sequences were fed to Long Short Term Memory networks (LSTMs) trained to discriminate between secure and insecure children. The main reason for using LSTMs is that they were shown to effectively model sequential information in a wide spectrum of problems. The number of neurons in the hidden layer is 128, a value that is commonly applied in the literature (no attempts were made with other values). The training was performed with an Adam optimizer [[41](#_ENREF_41)] and each iteration of the process includes two steps, namely estimate of the gradients (how the output of the network changes upon a change of its parameters) and update of the parameters (modification of the parameters that results into a better satisfaction of the training criterion). The learning rate (a numerical factor that modulates the size of the parameter change from one iteration to the other) has been set to $2\times10^{-4}$, a standard value commonly applied in the literature (see. e.g., Arjovsky et al., 2016) [[42](#_ENREF_42)], and no other values were tried.

The LSTMs were trained over small disjoint subsets of the training set, the mini-batches, the union of which corresponds to the entire training set. Such a strategy, known as mini-batch strategy [[43](#_ENREF_43)], aims at dealing with the computational issues resulting from the use of large amounts of training data. In the experiments of this work, every mini-batch includes 800 input sequences and the number of training epochs (iterations through gradient estimate and weight update steps mentioned above) is 30. The overall experimental setup corresponds to a leave-one-child-out strategy, meaning that a model is trained over data from all available children except one that is used as test set. Such a process is then repeated as many times as there are children and, at every iteration, a different child is left-out as a test. The main drawback of the leave-one-child approach is that it is difficult to estimate the generalization effectiveness of the trained models. However, the important advantage is that it is possible to exploit the full potential of the data at disposition and to estimate the performance that can be achieved when the entire available data can be used for training.

**References**

40. Gers FA, Schraudolph NN, Schmidhuber J. Learning precise timing with LSTM recurrent networks. Journal of machine learning research. 2002;3(Aug):115-43.

41. Kingma DP, Ba J. Adam: A method for stochastic optimization. arXiv preprint arXiv:14126980. 2014.

42. Arjovsky M, Shah A, Bengio Y, editors. Unitary evolution recurrent neural networks. International Conference on Machine Learning; 2016: PMLR.

43. Konečný J, Liu J, Richtárik P, Takáč M. Mini-batch semi-stochastic gradient descent in the proximal setting. IEEE Journal of Selected Topics in Signal Processing. 2015;10(2):242-55.
